# Depression and Anxiety in Cardiac Disease, diagnosing and screening

**DOI:** 10.1192/j.eurpsy.2023.1634

**Published:** 2023-07-19

**Authors:** T. Jupe, K. Provi, B. Zenelaj, E. Myslimi, I. Giannopoulos

**Affiliations:** 1 Psychiatric Hospital of Attica, Athens, Greece; 2 National Center for Children Treatment and Rehabilitation; 3 Freelancer Psychiatrist, Tirane, Albania

## Abstract

**Introduction:**

Among patients with heart disease, such as coronary artery disease or heart failure, depression and anxiety disorders are extremely common. In these populations, 20% to 40% have elevated depressive symptoms, and 15% to 20% suffer from MDD. Anxiety may be even more common than depression. A recent meta-analysis suggests that over 50% of patients with heart failure have elevated rates of anxiety, and 13% meet criteria for an anxiety disorder. These prevalence rates are significantly higher than those in the general population and highlight the high-risk status of cardiac patients for these disorders.

**Objectives:**

The aim of this study is to highlight the frequency of anxiety and depression in patients with cardiac health problems and to explain the mechanism by which anxiety and depression influence the manifestation of cardiac diseases.

**Methods:**

Α bibliographical review was performed using the PubMED platform. All relevant articles were found using the keywords: depression, anxiety, cardiac disease.

**Results:**

The links between depression, anxiety, and cardiovascular disease are complex and involve psychological, biological, and behavioral mechanisms. Depression, arrhythmias, and coronary artery disease frequently co-occur because they share common behavioral and pathophysiological drivers-unhealthy lifestyle, autonomic dysregulation, hypothalamic-pituitary-adrenal (HPA) axis dysregulation, endothelial dysfunction, and inflammation-that are intricately related to one another.

**Conclusions:**

In patients with cardiovascular disease, depression and anxiety disorders are common, persistent, and associated with poor functional and cardiac outcomes. As such, making a timely and accurate clinical diagnosis using DSM-5 criteria is critical. Safe and effective treatments are available for the management of these disorders in patients with cardiac disease, and it is hoped that such treatment can improve psychiatric health, quality of life, and medical outcomes.

**Disclosure of Interest:**

None Declared

